# The regulatory and scientific context in the study of the medicinal use of cannabis in Peru

**DOI:** 10.3389/fmed.2025.1598227

**Published:** 2025-10-09

**Authors:** Pedro Wong-Salgado, Jeel Moya-Salazar, Hans Contreras-Pulache, Claudio Pardo-Villarroel

**Affiliations:** ^1^Centro de Estudios de Cannabis, Lima, Peru; ^2^CANNAVITAL Clinica Especializada en Terapias con Cannabis, Lima, Peru; ^3^Faculty of Medicine, Universidad Señor de Sipán, Chiclayo, Peru; ^4^Faculty of Medicine, Universidad Privada Norbert Wiener, Lima, Peru; ^5^Laboratorio de Planificación Territorial, Departamento de Ciencias Ambientales, Facultad de Recursos Naturales, Universidad Católica de Temuco, Temuco, Chile

**Keywords:** cannabis, medicinal regulation, Peru, scientific research, cannabinoids

## Abstract

Cannabis has been historically utilized for medicinal purposes worldwide. However, it was classified as a controlled substance under the United Nations’ 1961 Single Convention on Narcotic Drugs, alongside coca and opium. This classification influenced many countries, including Peru, to adopt prohibitionist policies, even though the convention allowed medicinal use, scientific research, and even horticulture. Recent decades have witnessed a renewed interest in the therapeutic potential of cannabis, leading countries like Canada to establish medicinal frameworks. In 2017, Peru passed Law 30681, legalizing cannabis for medicinal use, scientific research, and pharmaceutical production through the obtention of licenses, and in 2021, Law 31312 allowed patient associations to grow and produce cannabis. Nevertheless, these regulations have proven insufficient, as no domestic production has taken place due to complex licensing requirements for both pharmaceutical companies and patient associations; the same scenario applies for scientific purposes. This article examines the regulatory challenges surrounding cannabis in Peru, focusing on scientific research. While the 2019 regulatory framework aimed to support medicinal access, it has fallen short in practice. Hard licensing process and stringent requirements have restricted scientific investigation, limiting it to observational studies and analysis of cannabis products available on the informal market. A recent study conducted between 2019 and 2023 analyzed native cannabis strains in four regions of Peru, highlighting the therapeutic potential of local cultivars. However, regulatory hurdles, such as sample obtention, transportation, and analysis, pose significant risks to researchers. The study suggests that Peru’s cannabis regulation requires urgent reforms, including simplifying licensing for scientific research, facilitating access for patients, and distinguishing personal cultivation from illicit drug trafficking. These changes are necessary to harness the medicinal potential of cannabis and support the development of local research and the possibility of local production.

## 1 Introduction

Cannabis is a worldwide cultivated medicinal plant with ancestral uses. However, along with cocaine, poppy, and a list of synthetic substances, it was included in the 1961 United Nations Single Convention on Narcotic Drugs (1) and subsequent control conventions (2). Although these conventions stipulate that its medicinal, scientific, industrial, and horticultural uses should not have been affected, some countries ultimately implemented substance control policies under a prohibitionist approach. Nevertheless, by the late 1990s, interest in the therapeutic potential of cannabis resurged, and by 2001, Canada became the first country in the Americas to establish a regulatory framework allowing its medicinal use (3–5). To date, over 40 countries worldwide have followed suit, including many classified as “high health surveillance countries” by the Peruvian Ministry of Health (6).

Although Peru approved Law 30681 (7) in 2017, permitting the medicinal and therapeutic use of cannabis, its regulation in 2019 (amended in 2024) only allowed pharmaceutical laboratories exclusive production rights. Additionally, Law 31312 (8) of 2021 allows patient associations to cultivate cannabis. However, the implementation of both laws has been insufficient, as no cultivation has taken place under these frameworks, neither by pharmaceutical companies nor by patient associations. As a result, all medicinal products commercially available through formal channels are derived from imported raw materials, driving up costs for patients. Furthermore, the process to obtain associative licenses is demanding, which leaves patients who choose cannabis horticulture unprotected against police intervention (9).

Similarly, actual regulation proposes a license for scientific research, lifting the prohibition on generating technical evidence in this area. However, the requirements are extremely bureaucratic, including security standards comparable to those required for pharmaceutical companies’ production, which discourage research. This situation limits the studies’ spectrum to those that do not require licenses, such as observational studies (patient follow-ups), knowledge and aptitude assessments, analyses of products from formal or informal markets, and collections of spontaneous or feral plant samples. It is in the last category that a recent study of our team demonstrated how samples of various cannabis cultivars found in four regions of Peru exhibit cannabinoid profiles with significant therapeutic potential (10). Conducted between 2019 and 2023, this research proposes a novel approach to the medicinal use of cannabis in Peru, focusing on pre-existing (informal) crops.

The objective of this work is to discuss the regulatory context of cannabis in Peru, highlighting the challenges in scientific research of this medicinal plant and proposing some regulatory modifications.

## 2 Historical aspects of regulation

The use of cannabis has been persecuted and stigmatized due to 1961 United Nations classification as “lacking of medicinal value” and “high potential of dependence” substance, in this way cannabis was considered harmful for public health in Peru since 1978, when the decree law 22095 on “repression of illicit drug trafficking” was enacted (11). This decree establishes penalties for the illicit trade of cannabis (and the other substances on the list) as criminal offenses. As it is expected, since that time, scientific interest has been more focused on harms and risks than on any therapeutic use of cannabis (12–14). Following the rediscovery of its medicinal properties, the number of scientific publications showing new evidence and confirming traditional uses with modern pharmacological basis has grown significantly in the last decade (15). This trend generates interest among healthcare professionals (16), but also skepticism among some conservative medical communities, such as psychiatry (17).

Cannabis (and its derivatives) was first scheduled under lists I and IV of control of the 1961 United Nations Single Convention on Narcotic Drugs. However, after a series of formal reviews by the World Health Organization (WHO) Expert Committee on Drug Dependence, in January 2019, six recommendations were proposed to modify the schedule status of cannabis (18), and in December 2020, the United Nations Commission on Narcotic Drugs (CND) voted on them. The unique recommendation adopted was the removal of cannabis and its derivatives from Schedule IV (the list of substances or plants deemed to have no medicinal value) (19). Thus, cannabis regained its ancestral status as a medicinal plant. Without further discussion, during this session, the Peruvian Ministry of Foreign Affairs voted against all WHO’s proposed modifications, including the one that was ultimately approved (20). By then, Peru had already enacted a law in 2017 and implemented it in 2019 permitting its medicinal use, research, and production (21).

**Figure 1:**
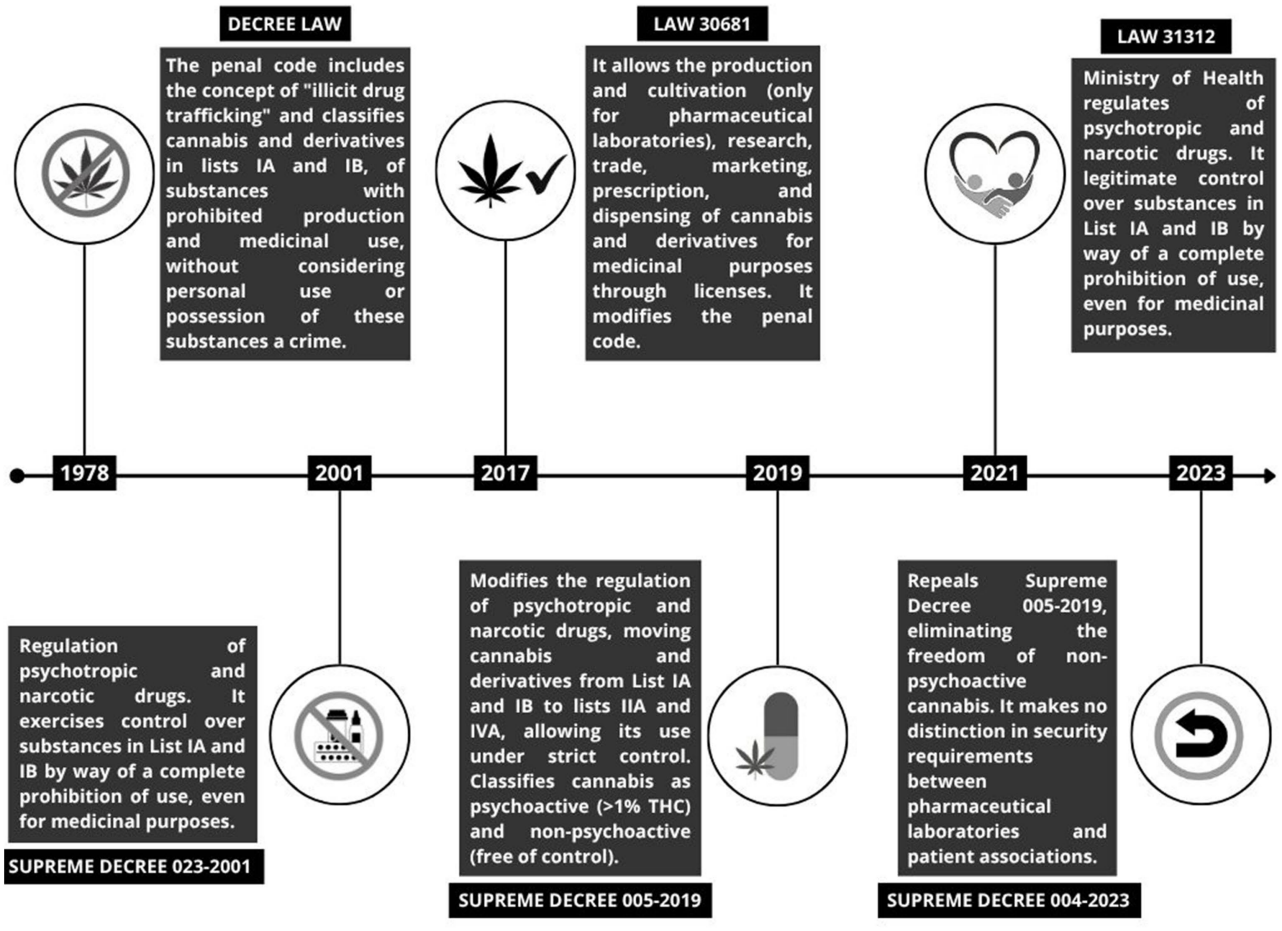
Timeline of cannabis regulation in Peru.

Since 2001, cannabis control policies in Peru have been implemented by the Health Ministry, prohibiting its complete medical use (prescription, dispensing, production, and scientific research) under imprisonment and professional disqualification (22). At the same time, non-medical (or adult) use and possession for personal use are explicitly not penalized under Article 299 of the current Penal Code (23). Nevertheless, in several cases, citizens are detained and charged under the “abstract offense” of micro-trafficking. Figure 1 shows the evolution of the regulation of cannabis in Peru in the last 50 years.

Seven years after the approval of the medicinal cannabis law, the results of evaluating its implementation (in terms of medicine availability) are not encouraging to the user community. Bureaucracy required by pharmaceutical laboratories and patient associations for cultivation and production has not been easy to fulfill in economic terms, of human resources and fragmentation of institutions that generate incompatible requirements. Consequently, legal cannabis supply is still imported, increasing costs for patients. Moreover, only 32 (0.11%) of the 28,133 pharmacies nationwide have Tetrahydrocannabinol (THC) available for dispensing to the nearly 50,000 officially registered patients (24). These results are consistent with previous media reports that show only 30% of patients acquire cannabis through a legal supply (25), leaving the rest of them at risk of the informal market or cultivating their plants, jeopardizing their freedom. It is precisely this underserved population that could, under medical supervision, benefit from the research of the therapeutic potential of Peru’s native cannabis cultivars.

## 3 The war on drugs: the case of cocaine and cannabis

The implementation of the decree law on illicit drug trafficking (1978) in Peru introduced a “prohibitionist” or “war on drugs” model, inspired by the United States’ approach to controlled substances (26). This model was enforced by the Ministry of the Interior and, indirectly, by the Ministry of Health, since the illicit trafficking of cannabis was considered a threat to public health. While Peru has been a key player in global cocaine production, ranking as the second-largest producer worldwide until 2021 (27), the same does not apply to cannabis, despite it remaining the most prevalent illicit substance. The differentiated regulatory treatment between the two substances persisted even though cocaine presents a higher toxicological risk than cannabis. This risk is reflected in its narrower safety margin (ratio between the effective dose and the lethal dose 50), as well as in its greater dependence potential compared to THC, the main psychoactive compound in cannabis (28), suggesting that risk assessment was not the sole basis for the formulation of these policies. Before the regulation of medical cannabis use in 2019 (D. S. 005-2019-SA), cocaine was permitted for medicinal purposes under Schedule IIA, whereas cannabis remained entirely prohibited under Schedules IA and IB. Even the National Commission for Development and Life without Drugs (DEVIDA), in its 2017–2021 strategy, identified two types of local demand for cannabis: for recreational use and for medicinal purposes (29).

## 4 Scientific research of cannabis in Peru

Following the government’s endorsement for research development in Peru through SUNEDU (National Superintendence of Higher Education) in 2018 and, under the protection of Law 30681 for cultivation for scientific purposes, there has been an initial appearance of studies in this field (30, 31). Though, this progress remains constrained by inadequate funding, ineffective research policies, and insufficient research interest among students (32, 33), which creates a challenging environment for advancing and ensuring the availability of medicinal research (34), particularly in the field of cannabinoids (Table 1).

**Table 1:** Transformation of conceptual definitions of cannabis in Peru.

Cannabis concept	Decree Law 22095 (1978)	Supreme Decree 004-2023 SA (regulation of Law 30681 of 2017), Law 31312 (2021) and Law 32195 (2024)
Definition	Art. 89, 5) “Cannabis” or “marijuana” means the dioecious plant that contains psychoactive principles	2.4. Cannabis: Flowering or fruiting tops of the cannabis plant (except for seeds and leaves not attached to the tops) from which the resin has not been extracted, whatever the name given to them. They are classified as: Psychoactive cannabis: Whose THC content is equal to or greater than 1% by dry weight and which are used for medicinal and therapeutic purposes Non-psychoactive cannabis: Whose THC content is less than 1% by dry weight and which are used for medicinal and therapeutic purposes. (Excluded from controlled medicines list)
Schedule	List “IA” in Legislative Decree 22095. Prohibition of trade for illicit trafficking Lists “IA” and “IB” in the narcotics regulations. Total prohibition for medicinal use	List “IIA” and “IVB” within the narcotics regulation Psychoactive cannabis needs special prescription and non-psychoactive cannabis needs simple prescription
Prescription	Punished from 2 to 5 years and loss of professional ability	Permitted, without restrictions on medical conditions or cannabinoid concentration
Cultivation or production	Punished from 8 to 15 years, if intended for illicit trafficking	Permitted for medicinal purposes under license, exclusively for pharmaceutical laboratories, public institutions and patient associations
Non-punishable possession	Adult use: 8 grams of inflorescences or 2 grams of extract for personal consumption or possession according to the penal code	Adult use: 8 grams of inflorescences or 2 grams of extract for personal consumption or possession according to the penal code Medical use: possession limit according to the medical prescription

In Peru, research related to cannabis cultivation or the use of imported extracts in medicine was prohibited until the enactment of the 2019 regulation. Since then, very few research projects have been permitted subject to obtaining a “scientific research license.” The requirements for this license are similar in quantity and complexity to those for pharmaceutical production. To date, the only pharmaceutical laboratory owner of a production license (which includes cultivation) has not fulfilled the requirements to produce medications from domestically cultivated cannabis (24). To understand these requirements, it is essential to note that the Peruvian justice system penalizes the illicit commercialization or trafficking of cannabis, whether on a large or micro scale, as a concrete offense. However, it also penalizes the cultivation of cannabis for personal use in a police intervention; these acts are considered by the prosecutor’s office to be related to illicit trafficking, an abstract offense. To the contrary, Article 299 of the Peruvian Penal Code states that the possession of 8 grams of plant material or 2 grams of its extracts is not punishable, but it does not clarify how to obtain them legally. In this way, as well as seed germination, as possession of cannabis plants for adult use puts citizens at risk of being accused of illicit trafficking or micro-trafficking. This situation creates potential discrimination against adult cannabis users during police interventions. Under this logic, the ownership of a license would allow citizens or companies to engage in concrete acts of production (cultivation and processing) and commercialization (national or international) of cannabis or its derivatives. Nevertheless, these scientific research licenses, which include cultivation (under the Law 30681) have proven to be difficult to obtain for local universities, discouraging academic interest and limiting the research development about the plant, which reflects a lack of studies in Peru.

Therefore, it can be inferred that scientific research exempt from license includes those who do not involve cultivation, production (processing), or commercialization (import or export), for example: (i) research using biomedical data from patients undergoing formal cannabis therapy, (ii) observations in adult users population, (iii) quality control studies of inflorescences or products from formal or informal markets, and other studies that do not involve direct interaction with the plant, such as literature or regulation reviews.

## 5 Chemotypes of Peruvian cannabis cultivars, testimony of our challenging research experience

In 2019, our team collected samples of inflorescences from local (spontaneous or feral) cannabis cultivars found in four regions of Peru. These samples were qualitatively and quantitatively analyzed with an analytical methodology previously performed (by a scientist of our team), looking for cannabinoid concentrations using gas chromatography (35) to predict their therapeutic potential based on current clinical literature.

The first challenge of this study was transporting the collected cannabis inflorescences samples from different regions of Peru to the Universidad Nacional Mayor de San Marcos (UNMSM) in Lima. We took samples from well-known areas of illegal cannabis cultivation and storage centers (27). We started asking local adult users knowledgeable of these areas who showed us local cultivars, leveraging our periodic participation in medical and adult cannabis user events since 2017, where we provided health interventions and collected data. Those people collaborated voluntarily with the research. However, transporting the samples, despite each region’s sample weighing approximately 600 mg dry, could have led to a “preventive” police intervention, risking the safety of the research team.

Until December 2019, when the qualitative analysis and subsequent cannabinoid quantification were conducted, no laboratory in Peru offered this service. This situation may be attributed to: (i) no quality control laboratory having developed an analytical technique for cannabinoid quantification due to limited academic or commercial interest, fear or stigma, (ii) the lack of analytical cannabinoid standards in Peru for sample comparison, as importing and possessing them was also a challenge for public or private parties, and (iii) the absence of skilled personnel in this field to perform the analytical technique. For this reason, we invited an expert analyst from Brazil to apply the gas chromatography technique and analyze the samples at the Toxicology Information, Control, and Environmental Management Support Center (CICOTOX) of the Faculty of Pharmacy and Biochemistry at UNMSM.

Until then, it was impossible to conduct proper cannabinoid quantification in Peru. Thus, scientific research in this area was limited to qualitative analysis, and quality control studies of formal or informal products were unfeasible. It was not until May 2022 that the National Health Institute (INS) of Peru made a cannabinoid quantification technique available to the public. However, as the costs are typically aimed at the pharmaceutical industry, they remain unaffordable for patient associations (36).

Proposals for improving the legal and regulatory framework for cannabis use in Peru.

Within the spectrum of regulatory improvements in legal and regulatory framework, the authors prioritize measures to facilitate patient access, promote productive activities, foster and encourage scientific interest in the plant, and prevent discriminatory practices against users. The following proposals are outlined:

(1)  **Update of the Narcotic and Psychotropic Drugs Regulation**: It is proposed to adopt a modern and realistic approach to the toxicological risk of controlled substances, with an emphasis on the constant evaluation of their impact on public health. In this context, the State and its institutions, particularly those dedicated to health, must prioritize unrestricted and bureaucracy-free access to these substances, ensuring that users are not hindered by unnecessary boundaries and that health professionals, both public and private, who prescribe medical cannabis are not subject to sanctions for such action. An example of this bureaucracy is the obligation to present a periodical prescription to access to a users’ association or to justify self-cultivation, without allowing public hospitals or community health centers to dispense prescriptions. This restriction could increase the associated costs, in addition to economically affecting medical consultations related to cannabis, in an eventual scenario of medical legalization, in which demand could increase.

  In Chile, according to Law 20.000 (Art. 8) on drugs, the use of medical cannabis requires a medical prescription, generally issued by a doctor in the private system. This generates an expenditure of more than 5% of the monthly minimum wage (approximately USD 25 in 2025) to acquire a prescription, which represents a considerable obstacle for the most economically vulnerable population. On the other hand, in Uruguay, according to Resolution No. 82/2020 in Act No. 227/2020 of the Institute for Regulation and Control of Cannabis (IRCCA), to justify cultivation for personal or collective use at home, it is sufficient to be over 18 years of age and register for free on the IRCCA platform, eliminating bureaucratic and economic barriers to access to medical cannabis and its derivatives. As for associations of medicinal cannabis users and pharmaceutical laboratories, while it is recognized that they must comply with rigorous controls given their role in large-scale production and distribution, it is crucial that these controls are not excessive and do not imply unnecessary obstacles. Protocols for production must be clear and aligned with international best practices to ensure product quality and safety.

(2)  **Formalizing Self-Provision Pathways for Cannabis**: Personal or associative cultivation for adult users should be formalized and explicitly included in the legislation, in order to avoid confusion between adult users and traffickers in cases of possession and/or use, in line with the approach proposed by the Brazilian government (37). According to Pardal (38), user associations in Uruguay have strict regulations limiting the amount of cannabis grown and distributed to members (up to 40 grams per month), so anyone who exceeds the limits without justification is breaking the law and may be suspected trafficker. That measure requires the enactment of a law recognizing the rights of adult users and self-provision of cannabis, accompanied by clarifications in the Penal Code to facilitate control and prevent illicit activities.
(3)  **Eliminating the Mandatory “Scientific Research License” for cannabis**: According to the Peruvian regulatory framework (Law 30681, 5°), the cultivation of cannabis for scientific purposes requires a specific license. The requirement for a scientific research license should be replaced by a simple registration system, similar to the existing chemical ingredients control registries D. S. N° 268-2019-EF and DL 1126. The implementation of these licenses currently faces economic and operational limitations that discourage investment, effort, and knowledge creation in this field, as pointed out by Buchman et al. for the case of Canada (39). This situation creates barriers for both researchers and industry, especially in contexts where costs and bureaucratic complexity are high. In addition to recognizing cultivation for medicinal and adult use purposes, it is also essential to advance in the recognition for scientific purposes, a necessary practice to achieve a higher level of knowledge and understanding of the plant in the country. It is important to note that current regulations in several countries, such as Canada, already allow research with cannabis under strict ethical and scientific controls, which facilitates the inclusion of this type of cultivation within a broader research framework (40). This change can be implemented through an amendment to Law 30681, which could streamline the administrative process without compromising current security and control standards.
(4)  **Promoting Multidisciplinary Research**: The current regulatory interpretation of the medical cannabis law limits scientific research to those areas strictly and exclusively related to its medicinal use. However, complementary disciplines such as botany, agronomy, geography, and phytochemistry are essential to understand the optimal conditions for growing and using this plant. For example, a groundbreaking 2012 study led by Evan Mills (41), who contributed to the Intergovernmental Panel on Climate Change (IPCC), co-recipient of the 2007 Nobel Peace Prize whit Al Gore, estimated that indoor cannabis production in the U. S. is associated with a carbon footprint of approximately 4.6 metric tons of CO_2_ per kilogram of cannabis produced. Subsequently, Summers et al. (42), using the Life Cycle Analysis (LCA) approach, reported emissions ranging between 2.2 and 5.2 metric tons of CO_2_ per kilogram, depending on geographic and technical conditions. These findings highlight the urgent need for multidisciplinary research on cannabis, beyond its medicinal and legal dimensions, especially considering the current global climate crisis.
(5)  **Utilizing Seized Cannabis Plants for Scientific Research**: Cannabis plants confiscated by the Peruvian National Police should be made available for scientific research through direct agreements between the Anti-Drug Directorate (DIRANDRO), universities and research centers. In this regard, it is worth noting the work of Gaoni and Mechoulam (43) on the isolation and synthesis of the active component of hashish (cannabis resin) from samples seized by the Israeli police, which was key to this discovery. In addition to this, other aspects must be incorporated, such as the ecological management of disposable waste, since current regulations require the mandatory incineration of seized materials and research by-products. This action can be implemented through a Ministerial Resolution of the Ministry of Interior, modifying the current directive on the “destruction of seized drugs,” and a Ministerial Resolution of the Ministry of Health, modifying the regulations on the medicinal use of cannabis.

## 6 Conclusion

Despite regulatory advancements in Peru since the approval of Law 30681 in 2017, access and research of cannabis remains limited. The complexity of procedures for obtaining cultivation, production, and research licenses has constrained local development, forcing patients to rely on costly imported products. Furthermore, the lack of accessible licenses for patient associations and the legal stigmatization of personal cultivation hinders adequate access. Scientific research faces significant barriers, discouraging the generation of crucial knowledge.

Moving forward, it is imperative to enhance controlled medicines policies and drug policies to simplify licensing requirements for production and research, incentivize local cultivation, and promote multidisciplinary research. For instance, repurposing seized plants for research would be a pragmatic measure to enhance local knowledge and reduce research costs. Additionally, ensuring that patients can access affordable medicinal cannabis through more inclusive and flexible regulatory processes is essential. These actions would contribute to the development of cannabis-based therapies in a more scientifically based, informed, and sustainable manner, ultimately improving the quality of life for thousands of patients.

## Data Availability

The original contributions presented in the study are included in the article/supplementary material, further inquiries can be directed to the corresponding authors.
